# VISTA functions as a protective immune checkpoint in indirect acute respiratory distress syndrome by modulating systemic and compartmentalized inflammation

**DOI:** 10.3389/fimmu.2025.1618135

**Published:** 2025-07-01

**Authors:** Baoji Hu, Chun-Shiang Chung, Chyna Gray, Yaping Chen, Jihong Jiang, Jianrong Guo, Alfred Ayala

**Affiliations:** ^1^ School of Gongli Hospital Medical Technology, University of Shanghai for Science and Technology, Shanghai, China; ^2^ Department of Anesthesiology, Shanghai Pudong Hospital, Fudan University Pudong Medical Center, Shanghai, China; ^3^ Division of Surgical Research, Department of Surgery, Rhode Island Hospital/The Warren Alpert School at Medicine at Brown University, Providence, RI, United States; ^4^ Department of Anesthesiology, Shanghai General Hospital, Shanghai Jiao Tong University School of Medicine, Shanghai, China; ^5^ Department of Anesthesiology, Shanghai Gongli Hospital, Naval Military Medical University, Shanghai, China

**Keywords:** VISTA, indirect ARDS, immune checkpoint, cytokine storm, myeloid cells, compartmentalized inflammation

## Abstract

**Background:**

Indirect acute respiratory distress syndrome (iARDS) is a life-threatening inflammatory lung injury often triggered by extrapulmonary insults. Although immune checkpoints are critical regulators of inflammation, the role of V-domain Ig suppressor of T-cell activation (VISTA) in iARDS remains unexplored.

**Methods:**

Using a murine model of iARDS, we compared outcomes in VISTA knockout (VISTA^−/−^) and wild-type mice. Disease severity was assessed through lung injury scoring, survival analysis, and cytokine/chemokine profiling in plasma, lung tissue, and peritoneal fluid. The therapeutic potential of VISTA was evaluated using an anti-VISTA antibody (13F3).

**Results:**

VISTA^−/−^ mice exhibited exacerbated lung injury, reduced survival, and elevated systemic levels of interleukin (IL)-6, IL-10, MIP-2, and KC compared to wild-type controls. While cytokine levels in lung tissue remained stable, peritoneal fluid mediators were dysregulated in VISTA^−/−^ mice, highlighting compartment-specific inflammatory regulation. Treatment with 13F3 reduced VISTA expression on myeloid and structural cells (monocytes, neutrophils, macrophages, epithelium, endothelium) and partially modulated cytokine/chemokine profiles across compartments.

**Conclusion:**

Our findings establish VISTA as a protective immune checkpoint in iARDS that restrains systemic hyperinflammation and organ damage. Although antibody-mediated VISTA targeting altered inflammatory pathways, its incomplete efficacy suggests complex, multifactorial mechanisms at play. These results position VISTA as a novel therapeutic target for iARDS and warrant further exploration of timed immunomodulatory strategies to harness its protective effects.

## Introduction

Acute respiratory distress syndrome (ARDS) manifests as sudden-onset respiratory failure characterized by severe hypoxemia and radiographic evidence of bilateral lung opacities (1). These symptoms arise from a spectrum of direct or indirect insults to the pulmonary parenchyma or vasculature (2). The most recent Berlin definition of ARDS outlines common risk factors, which are categorized as direct factors—such as pneumonia or aspiration of gastric contents—and indirect factors—including nonpulmonary sepsis, major trauma, and pancreatitis (3). Understanding the distinction between direct and indirect causes of ARDS is crucial in diagnosing and managing this life-threatening condition.

Immune checkpoint therapies, targeting negative checkpoint regulators (NCRs) such as anti-cytotoxic T-lymphocyte-associated antigen 4 (CTLA-4) and anti-programmed cell death 1 (PD-1), which block T-cell inhibitory pathways, have provided clinical benefit to a substantial number of patients (4). However, treatment of advanced melanoma with combined PD-1/CTLA-4 blockade commonly causes serious immune-mediated complications, including pneumonitis, myocarditis, and encephalitis (5, 6). Avoiding these complications has made treatment decisions more complex, requiring a balance between individual risk and tolerance for adverse reactions versus the superior clinical responses achieved with dual PD-1/CTLA-4 therapy (7, 8). VISTA, a more recently discovered NCR, is widely expressed (9) and plays roles in regulating naïve T-cell quiescence and sustaining peripheral tolerance (10). It also suppresses tumor aggression and burden in acute myeloid leukemia (11). Additionally, VISTA has been identified as a potential mediator of resistance to anti-PD-1 and anti-CTLA-4 immunotherapies in patients (12). However, therapeutic opportunities targeting VISTA have been limited by a lack of understanding of VISTA’s counter-receptor and function (13). We found critical inflammatory and physiological changes contributing to ARDS, with elevated VISTA expression on immune and parenchymal cells indicating its central role in lung injury (14). To better understand the immune profile of ARDS and investigate potential compensatory inhibitory pathways that may arise in the setting of immune checkpoint monotherapy, we conducted gene knockout and antibody-based experiments in mice.

## Materials and methods

### Animals and model induction

Eight- to twelve-week-old male C57BL/6J (The Jackson Laboratory, Bar Harbor, ME, USA) and VISTA knockout mice (15) were maintained in our animal care facility. All experiments were conducted in accordance with NIH *Guidelines for Animal Use and Care* and as approved by the Rhode Island Hospital Institutional Animal Care and Use Committee (Providence, RI; AWC#0040-16). Of note, male mice were used in this study because our laboratory has previously observed sex-based differences in immune responses to hemorrhage (Hem) and/or cecal ligation and puncture (CLP), with males being significantly more sensitive than pro-estrus females (16, 17). In other words, to avoid overlooking the potential effects of Hem/CLP due to the known protective influence of the female sex, we selected male mice, which are more susceptible to developing experimental ARDS.

Indirect ARDS (iARDS) was induced using a two-insult mouse model involving an initial hemorrhagic shock and a septic challenge (18). Mice were anesthetized in the atmosphere of servofluorane during the procedure. For the Hem protocol, polyethylene tubing (PE-10, 0.28 mm inner diameter × 0.64 mm outer diameter) was inserted into the femoral artery under an optical microscope. One side was used for drawing blood, and the other side for monitoring blood pressure. The volume of drawn blood was recorded and stopped once the blood pressure dropped to 35 mmHg ± 5 mmHg and was maintained at that level for 90 min. Following this, four times lactated Ringer’s solution (relative to the volume of blood drawn) was administered through the tube for resuscitation. The femoral arteries were then tied and sutured after resuscitation. In the sham group, the femoral arteries were tied and sutured without blood withdrawal or resuscitation. Mice were housed in the animal care facility with free access to food. At –24 h post-Hem, a secondary insult was induced by polymicrobial sepsis using the CLP method, which creates a consistent and reproducible septic focus. In the sham group, the abdomen was incised and sutured without CLP. Mice were euthanized at –24 h post-CLP, and lung tissues and blood samples were collected for further analysis.

### Treatment groups

WT mice in the treatment group were subcutaneously injected on the back with a hamster monoclonal antimouse VISTA antibody (13F3) (10 μg/g mouse body weight, 45 μL; clone 13F3, Bio X Cell Inc, Hanover, NH, USA.) after Hem and CLP, respectively. The 13F3 was diluted in saline. Mice in the control group received the same volume of nonspecific hamster IgG diluted in saline.

### Flow cytometric analysis

The expression levels of VISTA on various cell types—including neutrophils, monocytes/macrophages, and endothelial and epithelial cells—were assessed 24 h after CLP surgery. Flow cytometry was used to analyze VISTA expression in both whole blood samples and single-cell suspensions derived from lung tissues. The lung tissues were processed using an enzymatic dissociation buffer to generate single-cell suspensions, following these steps: (1) Prepare 10 mL of dissociation medium by adding 1 mL of collagenase/hyaluronidase and 1.5 mL of DNase I solution (1 mg/mL) to 7.5 mL of RPMI 1640 medium. Warm the solution to room temperature (15°C–25°C). (2) Harvest lung tissue into a tube containing PBS with 2% FBS. (3) Transfer the lung tissue to a dish without medium and mince it into a homogenous paste (< 1 mm in size) using a razor blade. (4) Transfer the minced lung tissue and the dissociation medium to a sterile 50-mL conical tube. Rinse the dish with the remaining 5 mL of dissociation medium and add it to the tube containing the minced tissue. Incubate at 37°C for 20 min using a Miltenyi^©^ GentleMACS Dissociator. Following digestion, the cell suspension was carefully filtered through a 70-mm cell strainer to obtain a homogenous single-cell mixture suitable for accurate flow cytometric analysis. The resultant cells were diligently counted using a microscope, and the concentration was adjusted to achieve a final cell density of 1 to 10 million cells per milliliter. The single-cell suspension was then stained, and fluorochrome expression was analyzed using a Miltenyi^©^ MACS Quant 10 flow cytometer (Miltenyi Biotec Inc., Auburn, CA, USA), as previously described in our laboratory (19, 20).

For flow cytometric analysis, debris (low FSC/SSC) and doublets (identified using FSC-H vs. FSC-W for singlet discrimination) were first excluded. Intact cells (moderate-to-high FSC/SSC) were then gated, followed by the exclusion of dead cells using a viability dye (e.g., PI). The harvested cells were stained with the following fluorochrome-conjugated antibody panel: anti-Ly6G (clone 1A8), anti-VISTA (clone MH5A), anti-CD11b (clone M1/70), anti-PD-1 (clone 135209), anti-CD31 (clone W18222B), anti-CD115 (clone T38-320), and anti-programmed cell death-ligand 1 (PD-L1; clone 124336). The cells were identified and quantified based on their expression of the following markers: CD11b^+^Ly6G^+^ (neutrophils), CD11b^+^F4/80^+^ (macrophages), CD31^+^ (endothelial cells), and CD115^+^ (epithelial cells).

### Histological analysis

Lung tissues were processed using hematoxylin and eosin (H&E) staining to assess morphological changes in these animals, as we previously described (20). Specimens from the lungs of experimental mice were harvested and fixed in formalin to preserve cellular details and prevent degradation. The fixed tissues were embedded in paraffin wax to provide support for sectioning. Thin sections (3–5 µm) were cut using a microtome and mounted on glass slides. The slides were then placed in xylene to dissolve the paraffin. The tissues were then rehydrated by passing the slides through a series of graded alcohol solutions (100%, 95%, 70%) and finally into distilled water. The slides were immersed in hematoxylin solution for 5–10 min. They were then rinsed in running tap water to remove excess stain. Bluing was performed by immersing the slides in ammonia water or lithium carbonate solution for 30 s to 1 min. The slides were subsequently in eosin solution for 1–3 min. Finally, the slides were passed through graded alcohol solutions (70%, 95%, 100%) to remove water. The tissue sections were cleared by immersing them in xylene, which rendered the tissue transparent. A few drops of mounting medium were applied to the tissue section. A cover slip was carefully placed over the section, ensuring that air bubbles were avoided. The slides were examined under a microscope to confirm optimal staining quality. As previously described by us, the extent of morphological changes in the lung specimens was assessed using a pathological scoring system. Specimens were scored from 0 to 4: 0 (normal), 1 (very mild impairment, < 25% of the field area), 2 (mild impairment, 25% to 50% of the visual field area), 3 (moderate impairment, 50% to 75% of the visual field area), and 4 (severe impairment, > 75% of the view area). Scoring was performed by two blinded pathologists based on alveolar wall thickening, interstitial edema, infiltration of inflammatory cells (such as neutrophils and macrophages), and the presence of hyaline membranes. Additionally, areas of atelectasis, Hem, and cellular debris may be observed, reflecting the pathological changes associated with the progression of ARDS (20, 21).

### Survival analysis

Survival analysis was conducted to assess the impact of VISTA on the survival of mice in the iARDS model. Mice were monitored daily for survival outcomes, and a Kaplan–Meier survival curve was generated to compare survival rates between wild-type and VISTA knockout mice. This analysis was used to evaluate the efficacy of VISTA in improving survival outcomes in the context of iARDS.

### Enzyme-linked immunosorbent assays

To quantify the levels of cytokines, including interleukin (IL)-6), IL-10, monocyte chemoattractant protein 1 (MCP-1), and tumor necrosis factor-alpha (TNF-α) (BD Biosciences, San Diego, CA, USA), as well as chemokines such as keratinocyte chemoattractant (KC) and macrophage inflammatory protein 2 (MIP-2) (R&D Systems, Minneapolis, MN, USA), enzyme-linked immunosorbent assays (ELISAs) were conducted according to the manufacturer’s instructions using lung tissue homogenates and plasma samples obtained from the experimental animals, as recently described by our laboratory (20). In brief, following the initial coating of the ELISA plate with the capture antibody, any excess unbound antibody was washed from the plate. Next, the unknown samples (lung tissue homogenates or plasma), along with diluted standard cytokine, were added. The samples were added in duplicate and at varying concentrations to ensure that their values fell within the detection range of the assay/standard cytokine curve. After sample incubation, excess material was again washed from the plate, and a biotinylated detection antibody was added. Finally, a biotin-binding chromogenic substrate was added to the plate. The antigen concentrations in the unknown samples were determined by comparing the optical density (OD) values to a standard curve generated using cytokines/chemokines of known concentrations, in accordance with the manufacturer’s instructions.

### Quantitative RT-PCR assays

To assess select changes in gene transcription, a concise version of the quantitative RT-PCR protocol was employed to detect mRNA expression of VISTA in lung tissue. RNA was isolated from preserved lung tissue using a suitable extraction kit (Takara Bio USA, Inc., San Jose, CA, USA). The extracted RNA was then converted to cDNA using a reverse transcription kit. The VISTA gene region was amplified from the cDNA using specific primers and a PCR master mix. cDNA synthesis was performed using a PrimeScript™ RT Reagent Kit (Takara Bio USA, Inc., San Jose, CA, USA). qRT-PCR was then performed using the ABIQ3 system (Applied Biosystems Corp., Waltham, MA, USA) and the SYBER PrimeScript™ RT Reagent Kit (Takara Bio USA, Inc., San Jose, CA, USA) according to the manufacturer’s recommendations. The following primer sequences (synthesized by Life Technologies Inc., Carlsbad, CA, USA) were used: murine VISTA 5′-CTC CTT GCT ATT TTC CTG GCT G-3′ and 5′-AGG TGA GGG TGG CAT TCT GT-3′; IL-6 5′-ATG GAT GCT ACC AAA CTG GAT-3′ and 5′-TGA AGG ACT CTG GCT TTG TCT-3′ (sense/antisense); TNF-α 5′-AGG CTC ATC CTT GCC TTT GTC TCT-3′ and 5′-TCA GCA GCT ACC CAC ACT TCA CTT-3′ (sense/antisense); IL-10 5′-CCA GTT TTA CCT GGT AGA AGT GAT G-3′ and 5′-TGT CTA GGT CCT GGA GTC CAG CAG ACT C-3′ (sense/antisense); MCP-1 5′-TCA CCT GCT GCT ACT CAT TCA CCA-3′ and 5′-AAA GGT GCT GAA GAC CTT AGG q′ (sense/antisense); MIP-2 5′-AAAGTTTGCCTTGACCCTGAA-3′ and 5′-TCTTTGGTTCTTCCGTTGAGG-3′ (sense/antisense); KC 5′-ACTGCACCCAAACCGAAGTC-3′ and 5′-TGGGGACACCTTTTAGCATCTT-3′ (sense/antisense); and 18s 5′-GGA CAC GGA CAG GAT TGA CAG ATT-3′ and 5′-AAT CGC TCC ACC AAC TAA GAA CGG-3′ (sense/antisense). Amplified products were quantified using real-time PCR analysis, employing either a fluorescent probe or SYBR Green. Duplicate CT values were analyzed using the comparative CT (ΔΔCt) method. The amount of target (2−ΔΔCt) was obtained by normalizing to GAPDH relative to a control (nonstimulated cells or sham mice). VISTA expression data were normalized to 18s, and relative expression levels were calculated (22).

### Statistical analysis

Data were presented as mean values with standard error of the mean (SEM). As the group sizes were typically fewer than 10 animals/group, a Kruskal–Wallis test was applied to determine whether statistically significant differences existed between group means. Statistical significance for survival analysis was assessed using log-rank tests. A *p*-value of less than 0.05 was considered indicative of a statistically significant difference between group means.

## Results

### The development of iARDS

**Figure 1:**
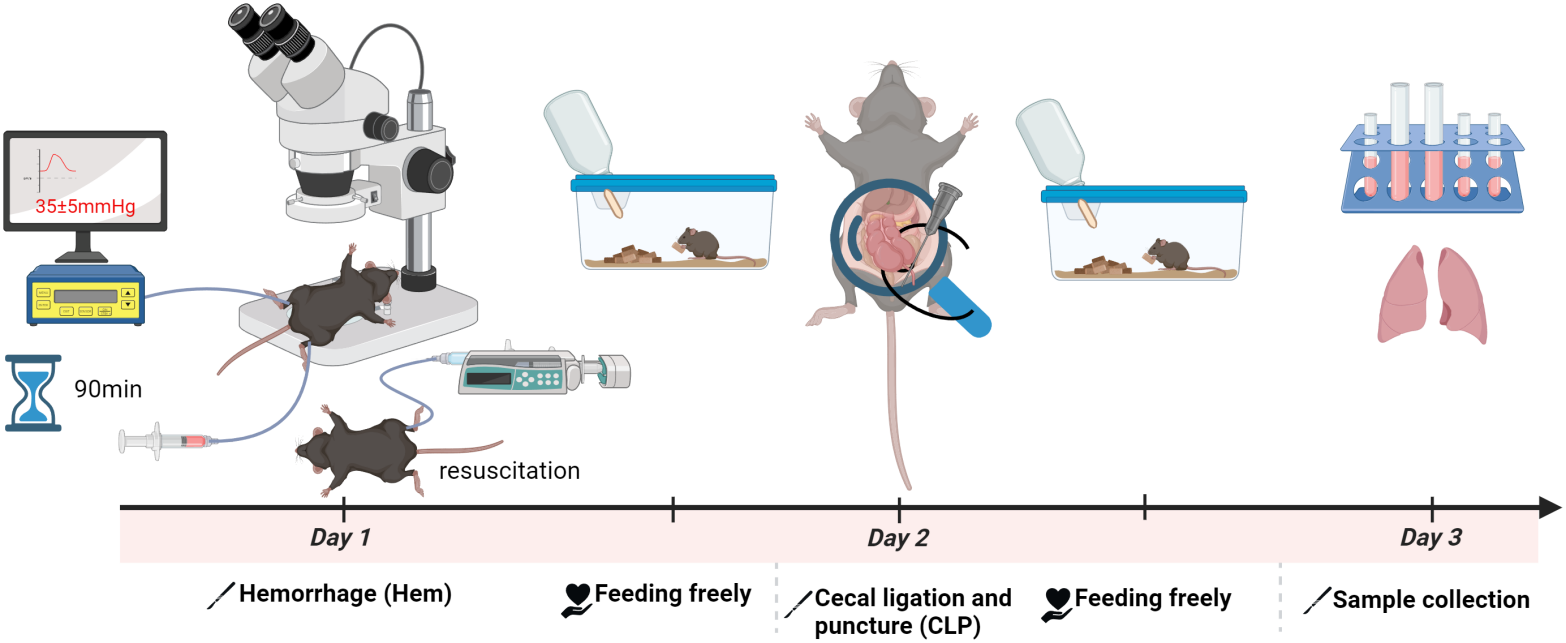
Schematic of the hemorrhagic shock (Hem)/CLP mouse model for studying iARDS. C57BL/6J mice were subjected to Hem on day 1, followed by CLP on day 2 to induce polymicrobial sepsis. This sequential Hem/CLP model recapitulates key features of hemorrhagic combined with septic shock, mimicking the pathophysiological progression of ARDS. The figure was created with BioRender.com.

To investigate the development of iARDS, we employed a “double-hit” mouse model that simulates septic shock (23). This approach involves HEM followed by sepsis induced by CLP—a sequence referred to as Hem/CLP. As depicted schematically in **Figure 1**, C57BL/6J mice first undergo Hem (day 1), followed by CLP (day 2).

### VISTA deficiency exacerbates mortality in a murine model of iARDS

To investigate the role of VISTA in iARDS, we established an experimental model using C57BL/6J (wild-type [WT]) and VISTA^−/−^ mice.

**Figure 2:**
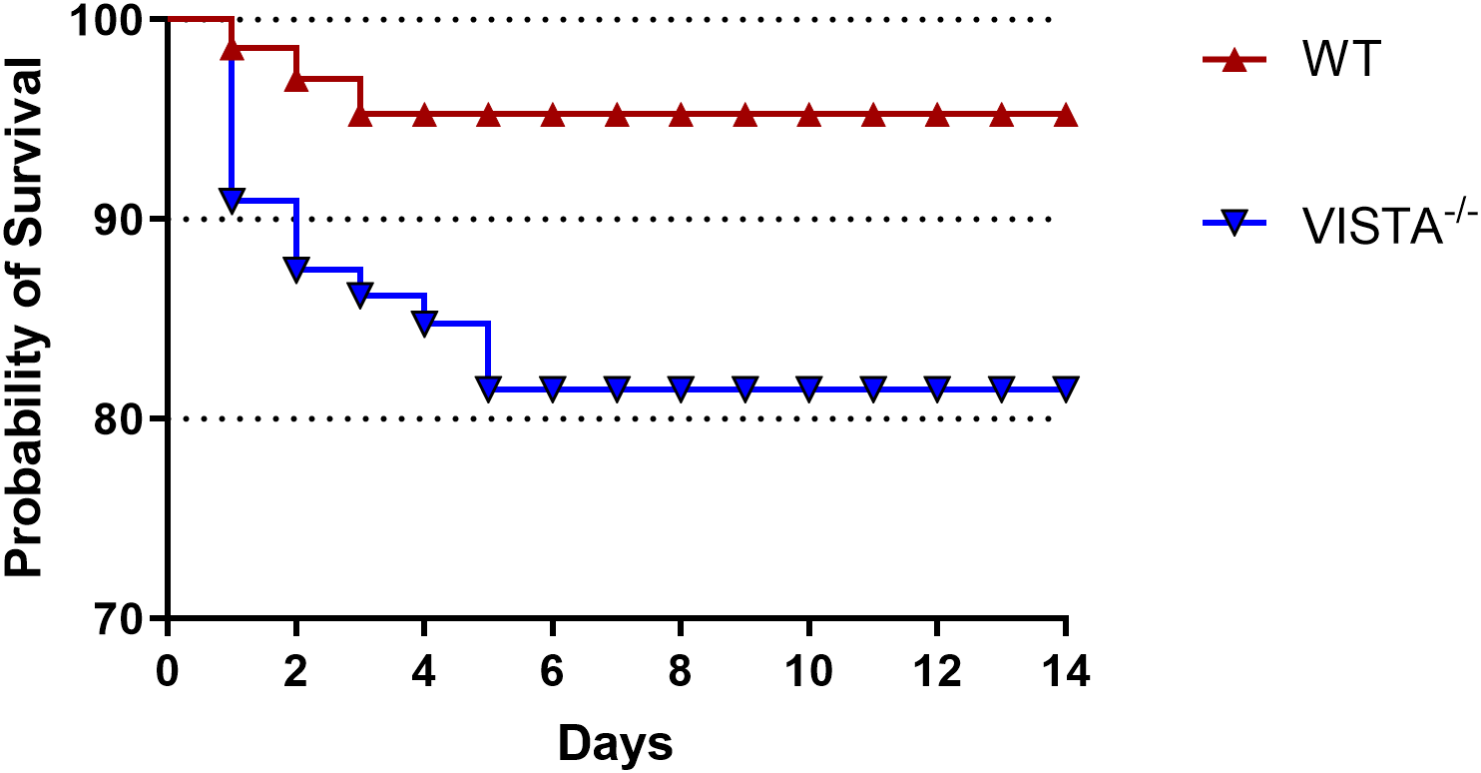
VISTA deficiency exacerbates mortality in the iARDS model. Survival curves of C57BL/6J wild-type (WT) and VISTA^−/−^ mice subjected to the Hem/CLP model over 14 days. VISTA^−/−^ mice exhibited significantly reduced survival compared to WT controls, highlighting VISTA’s critical role in suppressing dysregulated inflammation and maintaining immune homeostasis during iARDS progression. These findings demonstrate that VISTA provides protection against lethal systemic inflammation and organ failure in iARDS. The number of animals in each group was 16 for WT and 15 for VISTA^−/−^; the *p*-value was determined by log-rank test (*p* < 0.05).

Over a 14-day follow-up period, we observed a significantly lower survival rate in VISTA^−/−^ mice compared to their WT counterparts. This striking difference in mortality underscores the protective role of VISTA in mitigating the severity of iARDS. Specifically, VISTA deficiency appears to exacerbate disease progression, leading to increased lethality. These findings are visually summarized in **Figure 2**, which depicts the survival curves for both groups.

### Enhanced immune cell infiltration and epithelial disruption in VISTA-deficient mice following Hem/CLP

To assess the impact of VISTA deficiency on lung tissue pathology, we performed histopathological analysis using optical microscopy. VISTA^−/−^ mice exhibited a marked increase in immune cell infiltration within lung tissue compared to WT mice. This infiltration was accompanied by loss of epithelial integrity—a hallmark of tissue damage—and compromised barrier function. These findings suggest that VISTA deficiency exacerbates lung injury by promoting immune cell recruitment and disrupting the structural organization of the lung epithelium.

**Figure 3:**
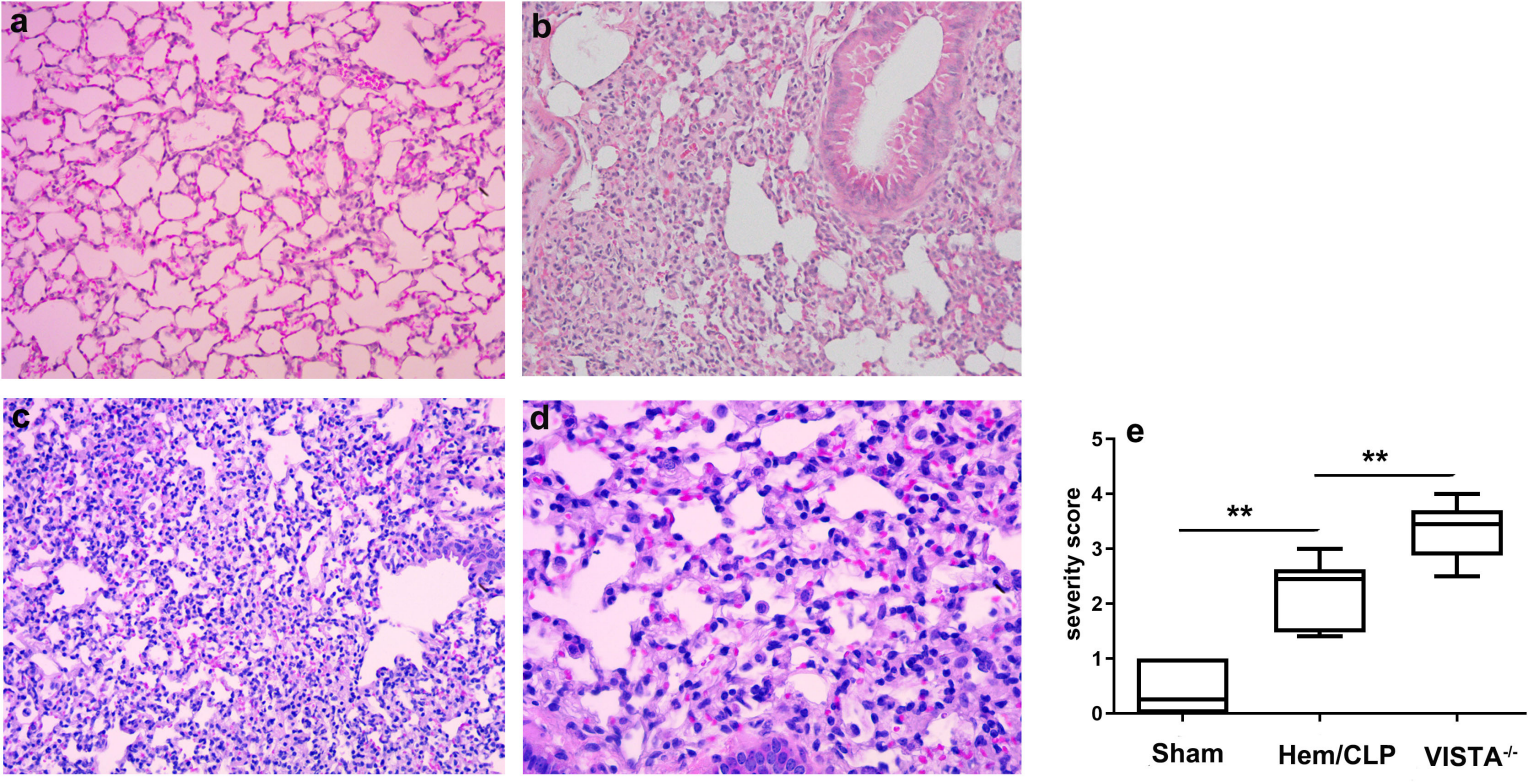
VISTA deficiency exacerbates histopathological lung injury and immune dysregulation in iARDS. Representative lung tissue sections from wild-type (WT) and VISTA^−/−^ mice post-Hem/CLP, stained with H&E. VISTA^−/−^ mice exhibited pronounced immune cell infiltration (neutrophils, macrophages, lymphocytes), loss of epithelial integrity, and elevated lung injury severity scores compared to WT controls. These findings demonstrate VISTA’s critical role in restraining inflammatory cell recruitment, preserving alveolar epithelial integrity, and mitigating tissue damage in ARDS. **(a)** Sham (WT, × 200 magnification), **(b)** Hem/CLP (WT, × 200 magnification), **(c)**, Hem/CLP (VISTA deficiency, × 200 magnification), **(d)** Hem/CLP (VISTA deficiency, × 400 magnification), **(e)** severity scores in different groups. The data in **(e)** are presented as the mean ± SEM. *p*-values were determined by the Kruskal–Wallis test. ^**^ *p* < 0.01.

Following Hem/CLP, lung tissue sections from VISTA^−/−^ mice revealed abundant infiltrating cells, including neutrophils, macrophages, and lymphocytes, indicative of an intense inflammatory response. In contrast, WT mice exhibited a more controlled and less severe immune cell influx. The severity score, quantifying the extent of tissue damage and inflammation, was significantly higher in VISTA^−/−^ mice compared to WT controls (*p* < 0.01), as illustrated in **Figure 3**. This elevated severity score underscores VISTA’s critical role in modulating inflammatory responses and maintaining tissue homeostasis.

### VISTA deficiency modulates inflammatory responses across compartments in a murine model of iARDS

**Figure 4:**
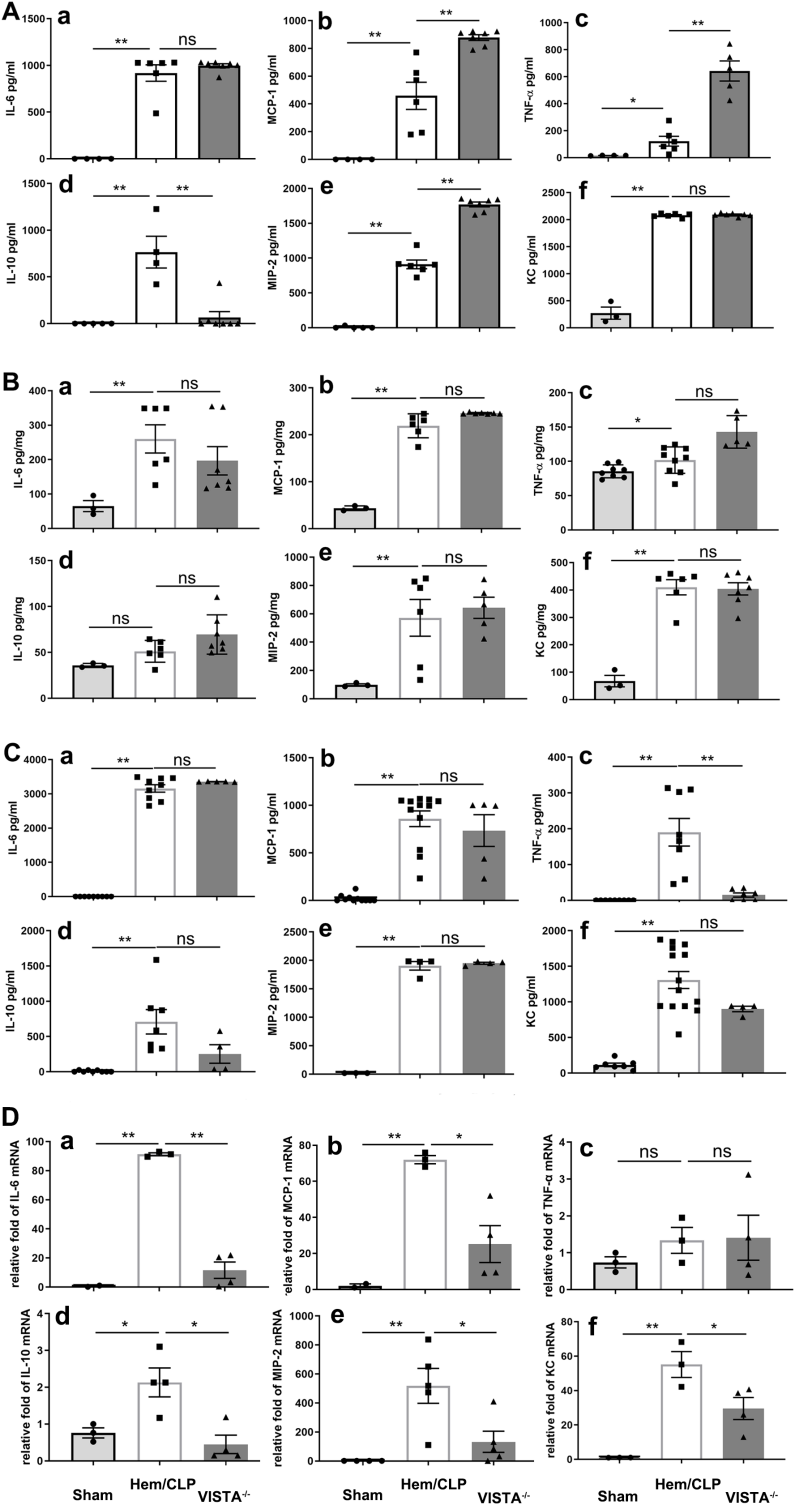
VISTA deficiency drives compartment-specific dysregulation of inflammatory mediators in iARDS. **(A)** Plasma cytokine/chemokine profiles (ELISA): VISTA^−/−^ mice exhibited elevated proinflammatory mediators (MCP-1, TNF-α, MIP-2) and reduced IL-10 compared to WT controls. **(B)** Lung tissue analysis: while Hem/CLP induced increases in cytokines/chemokines (MCP-1, TNF-α, MIP-2, IL-6, IL-10, KC) in WT mice, these responses were blunted in VISTA^−/−^ mice. **(C)** Peritoneal fluid: cytokine levels (MCP-1, IL-6, IL-10, MIP-2, KC) remained unchanged in VISTA^−/−^ mice, while others were suppressed. **(D)** mRNA quantification (qPCR): VISTA^−/−^ mice displayed transcriptional suppression of MCP-1, MIP-2, IL-6, IL-10, and KC, while TNF-α mRNA levels remained unchanged, suggesting potential posttranscriptional regulation. Data highlight VISTA’s role in restraining systemic hyperinflammation (plasma) while maintaining tissue-specific immune equilibrium (lung/peritoneum), underscoring its critical immunoregulatory function in iARDS pathogenesis. **(a)** IL-6, **(b)** MCP-1, **(c)** TNF-α, **(d)** IL-10, **(e)** MIP-2, **(f)** KC. The data are presented as the mean ± SEM. *p*-values were determined by the Kruskal–Wallis test. ^*^ *p* < 0.05; ^**^ *p* < 0.01; ns, no significance.

To elucidate the impact of VISTA on the pathogenesis of inflammatory iARDS, we measured plasma levels of proinflammatory cytokines and chemokines using ELISA. In VISTA^−/−^ mice, we observed a postprocedural surge in the cytokines MCP-1 (*p* < 0.01) and TNF-α (*p* < 0.01), as well as the chemokine MIP-2 (*p* < 0.01), compared to WT mice. Concurrently, the anti-inflammatory cytokine IL-10 (*p* < 0.01) was significantly suppressed in VISTA^−/−^ mice, as depicted in **Figure 4A**. This heightened proinflammatory state in the plasma suggests that VISTA deficiency exacerbates systemic inflammation, potentially contributing to the onset of iARDS.

Expanding our analysis to lung tissues, we found that levels of MCP-1 (*p* < 0.01), TNF-α (*p* < 0.01), MIP-2 (*p* < 0.01), IL-6 (*p* < 0.01), and KC (*p* < 0.01) increased significantly following Hem/CLP in WT mice. However, in VISTA^−/−^ mice, these mediators did not exhibit significant changes compared to WT controls, as illustrated in **Figure 4B**. This divergence between plasma and lung tissue responses underscores the compartment-specific effects of VISTA deficiency, with systemic inflammation predominating in plasma but a muted response in the lung microenvironment.

In peritoneal fluid, most cytokines and chemokines were reduced in VISTA^−/−^ mice, except for MCP-1, IL-6, IL-10, MIP-2, and KC, which remained unchanged compared to WT mice (**Figure 4C**). These findings further underscore the tissue-specific nature of VISTA-mediated immune regulation, with distinct inflammatory profiles observed across different biological compartments.

Intriguingly, analysis of mRNA expression revealed significant suppression of MCP-1 (*p* < 0.01), MIP-2 (*p* < 0.01), IL-6 (*p* < 0.01), IL-10 (*p* < 0.01), and KC (*p* < 0.01) in VISTA^−/−^ mice, indicating a dynamic molecular response at the transcriptional level. In contrast, TNF-α mRNA expression remained unchanged, suggesting a more nuanced regulatory mechanism potentially involving posttranscriptional or posttranslational modifications. These findings, visually depicted in **Figure 4D**, provide further insight into the molecular underpinnings of the observed biological response.

### Significant suppression of VISTA expression across immune and structural cells

**Figure 5:**
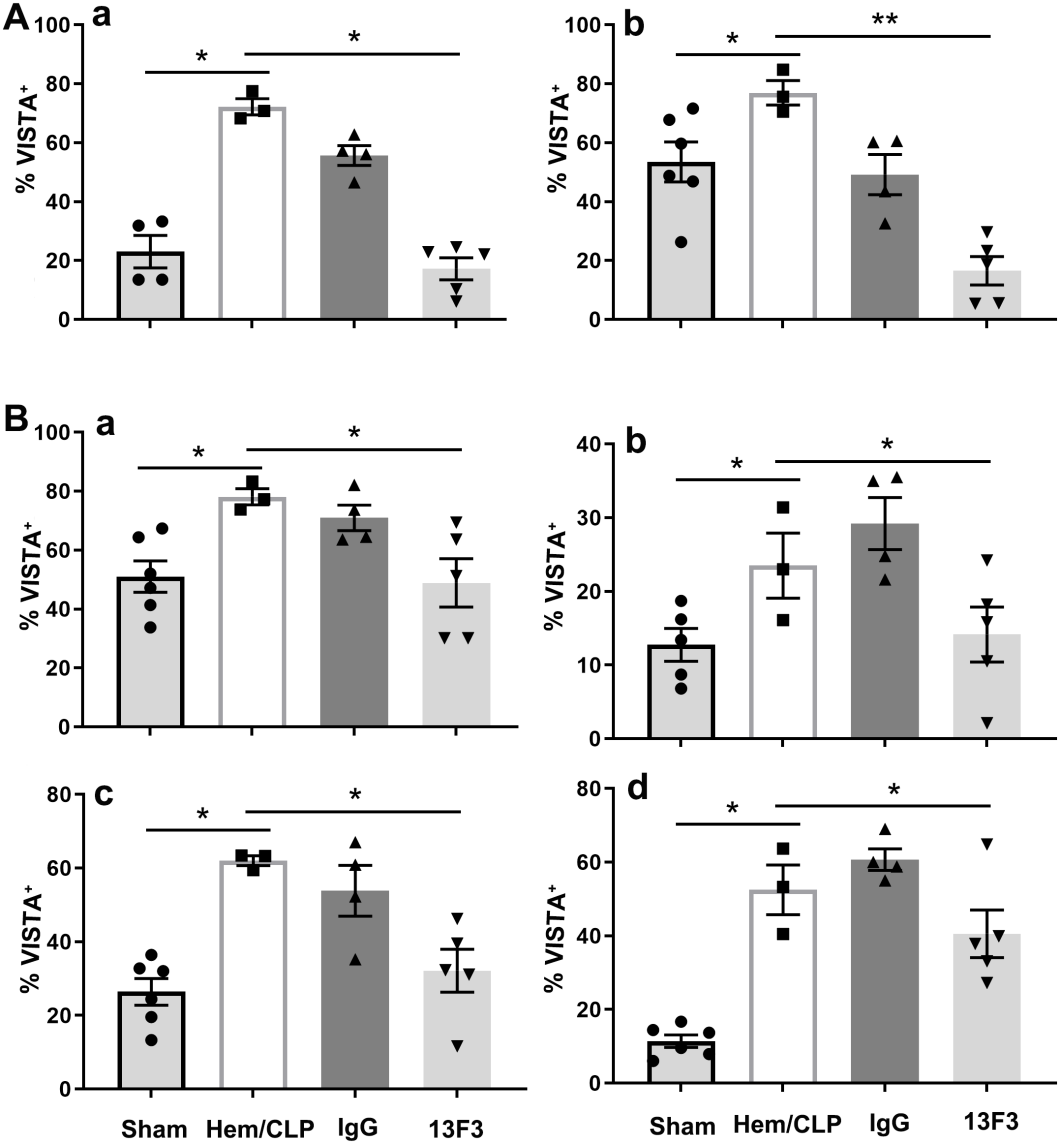
13F3 suppresses VISTA expression (% VISTA^+^) across immune and structural cell populations in iARDS. **(A)** Flow cytometry analysis showed that 13F3 treatment significantly reduced VISTA surface levels on peripheral blood monocytes, as shown by the percentage of cells that were positive for VISTA (% VISTA^+^). In peripheral blood monocytes and neutrophils, 13F3 treatment significantly reduced VISTA surface levels. **(B)** Lung tissue analysis: 13F3 downregulated VISTA expression on macrophages, neutrophils, epithelial cells, and endothelial cells, indicating broad checkpoint inhibition. This pan-cellular suppression of VISTA likely disrupts immune tolerance pathways and enhances anti-inflammatory responses in both circulating and tissue-resident cells. The data suggest that 13F3 reprograms the lung microenvironment by attenuating VISTA-mediated immunosuppression, offering therapeutic potential for ARDS. **(a)** Monocytes/macrophages, **(b)** neutrophils, **(c)** epithelial cells, **(d)** endothelial cells. The data are presented as the mean ± SEM. *p*-values were determined by the Kruskal–Wallis test. ^*^ *p* < 0.05; ^**^ *p* < 0.01; ns, no significance.

To investigate the impact of 13F3 on VISTA, a critical immune checkpoint molecule, we measured its expression on various cell populations using flow cytometry. In peripheral blood, VISTA expression was assessed on monocytes (*p* < 0.05) and neutrophils (*p* < 0.01), two key innate immune cell types. As shown in **Figure 5A**, 13F3 treatment significantly suppressed VISTA expression on both monocytes and neutrophils. This downregulation suggests that 13F3 may modulate immune checkpoint activity in circulating immune cells, potentially enhancing their responsiveness to inflammation.

The analysis was extended to lung tissue, where VISTA expression was evaluated on a broader range of cell types, including macrophages (*p* < 0.05), neutrophils (*p* < 0.05), epithelial cells (*p* < 0.05), and endothelial cells (*p* < 0.05). As shown in **Figure 5B**, VISTA expression was significantly suppressed across all these cell populations following 13F3 treatment. This widespread downregulation highlights the systemic effects of 13F3 on immune checkpoint regulation within the lung microenvironment.

The suppression of VISTA on macrophages and neutrophils in lung tissue aligns with findings in peripheral blood, reinforcing the consistency of 13F3’s effects on innate immune cells. Additionally, the reduced VISTA expression on epithelial and endothelial cells suggests that 13F3 may also influence the immune-regulatory functions of structural cells, which are critical for maintaining tissue homeostasis and modulating immune responses.

These findings collectively underscore the broad and potent ability of 13F3 to suppress VISTA expression across diverse cell types, both in circulation and within tissues. Given VISTA’s role in inhibiting T-cell activation and promoting immune tolerance, its downregulation by 13F3 may have significant implications for modulating anti-inflammatory actions.

### Antimouse VISTA neutralizing antibody (13F3) exerts compartment-specific immunomodulatory effects in ARDS through differential regulation cytokine/chemokine networks

**Figure 6:**
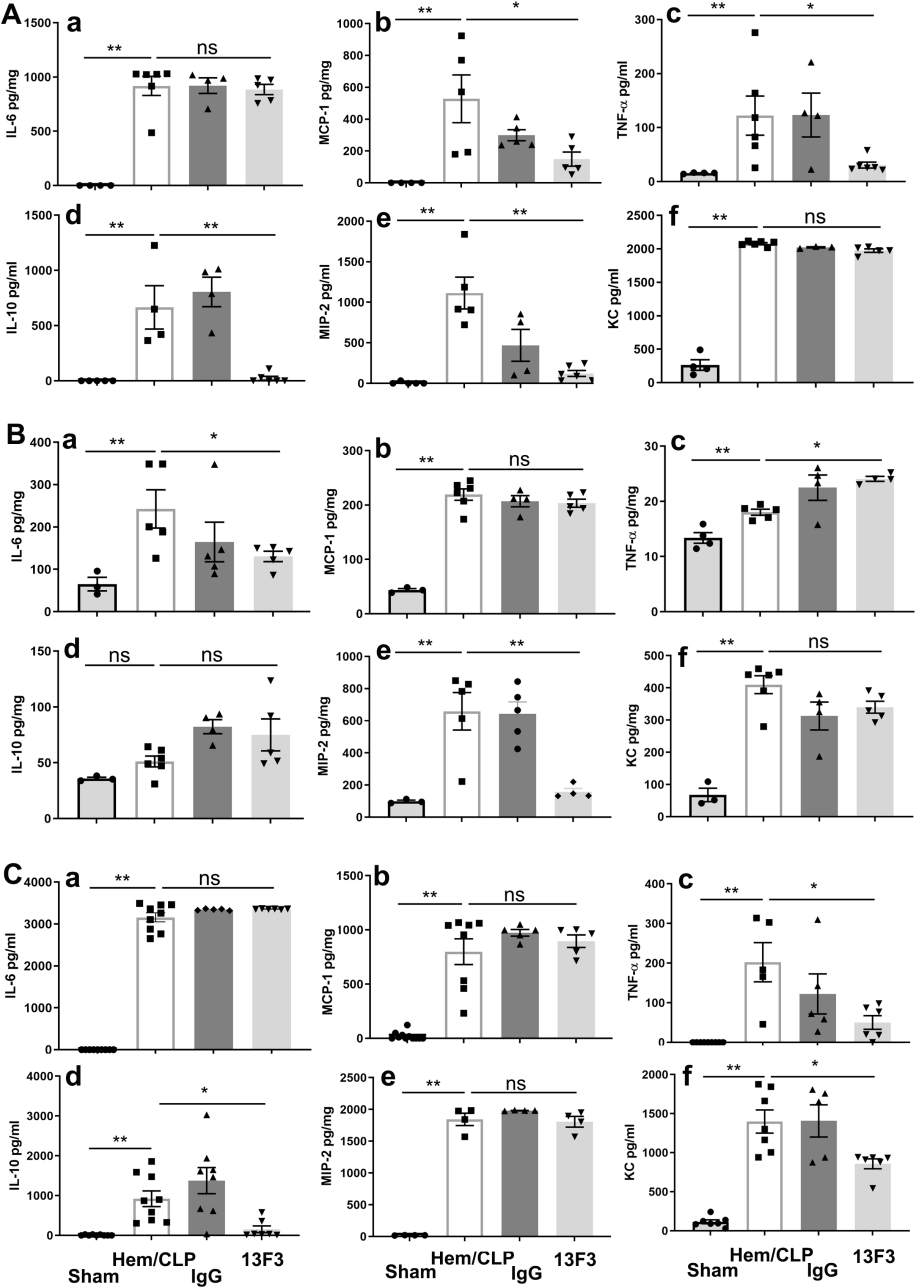
13F3 exerts compartment-specific immunomodulatory effects in ARDS by differentially regulating cytokine/chemokine networks (ELISA). **(A)** Plasma analysis: 13F3 suppressed proinflammatory mediators (MCP-1, TNF-α, MIP-2) and anti-inflammatory IL-10, indicating systemic mitigation of cytokine storm. **(B)** Lung tissue: 13F3 reduced IL-6 and MIP-2 but paradoxically elevated TNF-α, suggesting tissue-specific NF-κB pathway modulation. **(C)** Peritoneal fluid: TNF-α and IL-10 mirrored plasma trends, while KC suppression highlighted neutrophil chemotaxis inhibition. Unchanged IL-6/MCP-1/MIP-2 levels imply compartmentalized regulation. Data reveal 13F3’s nuanced immunomodulation—taming systemic inflammation while eliciting context-dependent responses in tissues—positioning it as a precision therapeutic for ARDS-driven hyperinflammation. **(a)** IL-6, **(b)** MCP-1, **(c)** TNF-α, **(d)** IL-10, **(e)** MIP-2, **(f)** KC. The data are presented as the mean ± SEM. *p*-values were determined by the Kruskal–Wallis test. ^*^ *p* < 0.05; ^**^ *p* < 0.01; ns, no significance.

The immunomodulatory effects of 13F3 were investigated by analyzing the expression of key cytokines and chemokines in different biological compartments. As demonstrated in **Figure 6A**, treatment with 13F3 led to significant suppression of the cytokines MCP-1 (*p* < 0.05), TNF-α (*p* < 0.05), and IL-10 (*p* < 0.01), as well as the chemokine MIP-2 (*p* < 0.01). These molecules are central to inflammatory and immune responses, and their downregulation suggests a potent anti-inflammatory effect of 13F3 in this context.

In lung tissue, as shown in **Figure 6B**, 13F3 treatment resulted in suppression of IL-6 (*p* < 0.05) and MIP-2 (*p* < 0.01), while TNF-α (*p* < 0.05) expression was notably elevated. This tissue-specific response highlights the complexity of 13F3’s effects, potentially reflecting differences in local immune microenvironments or signaling pathways.

Interestingly, in peritoneal fluid, the cytokines TNF-α (*p* < 0.05) and IL-10 (*p* < 0.05) remained suppressed following 13F3 treatment, consistent with the findings in **Figure 6A**. However, IL-6, MCP-1, and MIP-2 exhibited no significant changes in expression. In contrast, the chemokine KC (*p* < 0.05) was significantly suppressed, as illustrated in **Figure 6C**. This differential regulation suggests that 13F3 exerts distinct effects depending on the tissue or fluid compartment, potentially reflecting variations in cellular composition or microenvironmental factors.

These findings collectively emphasize the tissue-specific and context-dependent nature of 13F3’s immunomodulatory activity. The selective suppression or enhancement of cytokine and chemokine expression by 13F3 across different compartments highlights its potential as a targeted therapeutic agent for modulating immune responses in inflammatory or disease settings.

### Antimouse VISTA neutralizing antibody (13F3) selectively modulates cytokine/chemokine mRNA expression and immune checkpoint signaling in iARDS

**Figure 7:**
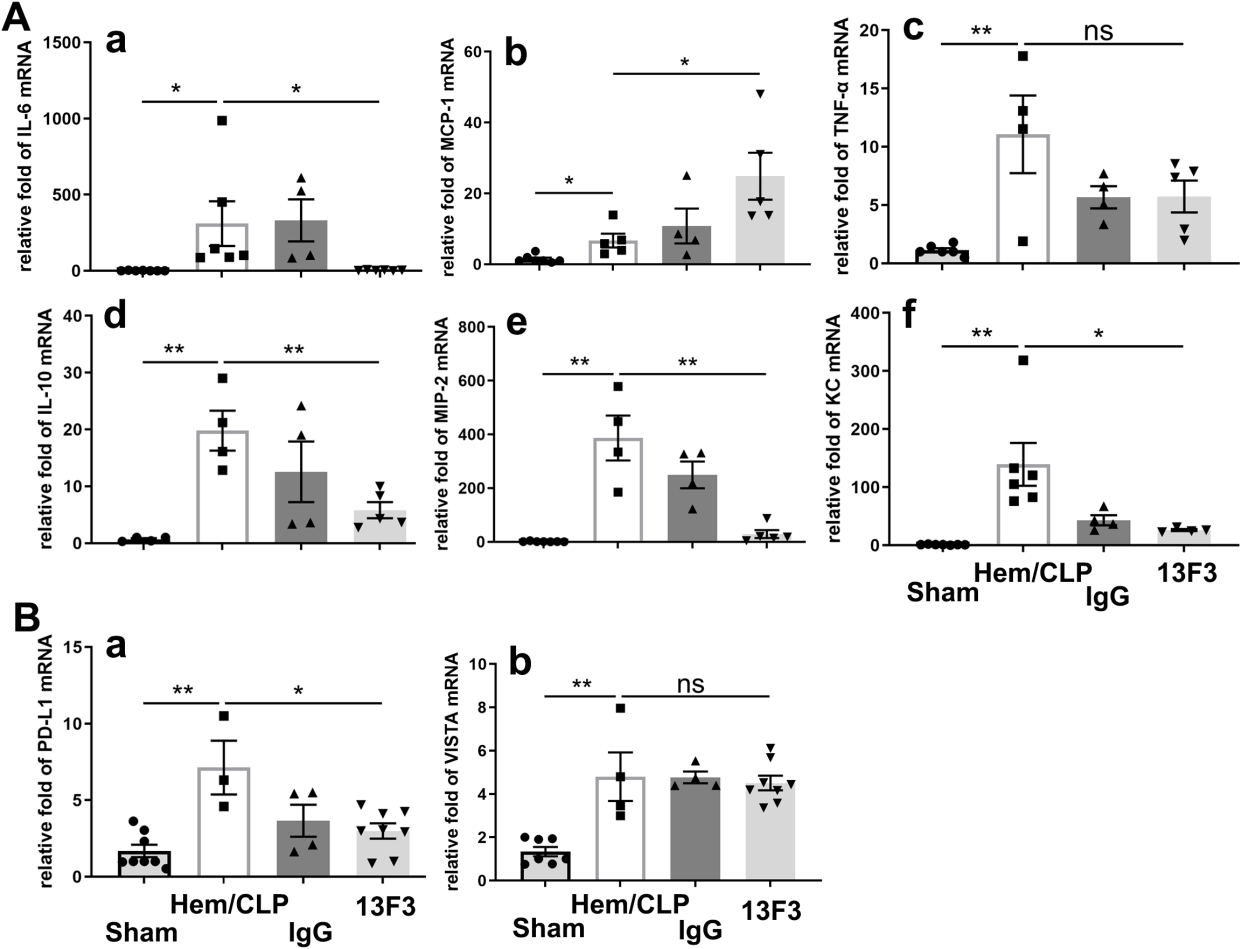
13F3 selectively modulates cytokine/chemokine mRNA expression and immune checkpoint signaling in ARDS. **(A)** qPCR analysis of inflammatory mediators: 13F3 suppressed mRNA levels of IL-6, IL-10, KC, and MIP-2, while amplifying MCP-1, indicating divergent regulation of chemokine networks. **(B)** Immune checkpoint profiling: 13F3 significantly downregulated PD-L1 mRNA (critical for T-cell inhibition) but left VISTA mRNA unaltered, highlighting its precision in targeting specific immunosuppressive pathways. These results suggest that 13F3 reprograms inflammatory transcription while selectively dismantling PD-L1-mediated immune evasion, positioning it as a dual-action therapeutic for ARDS and immune dysregulation. **(a)** IL-6, **(b)** MCP-1, **(c)** TNF-α, **(d)** IL-10, **(e)** MIP-2, and **(f)** KC in **(A)**; **(a)** PD-L1 and **(b)** VISTA in **(B)**. The data are presented as the mean ± SEM. *p*-values were determined by the Kruskal–Wallis test. ^*^ *p* < 0.05; ^**^ *p* < 0.01; ns, no significance.

As illustrated in **Figure 7A**, treatment with 13F3 suppressed mRNA expression of IL-6 (*p* < 0.05), IL-10 (*p* < 0.01), KC (*p* < 0.05), and MIP-2 (*p* < 0.01), all of which play pivotal roles in inflammation and immune regulation. In contrast, the expression of MCP-1 (*p* < 0.05), a key chemokine involved in monocyte recruitment, was notably amplified by 13F3, suggesting selective modulation of chemokine signaling pathways.

A particularly intriguing observation was the significant suppression of PD-L1 (*p* < 0.05) mRNA expression by 13F3, as depicted in **Figure 7B**. PD-L1 is a critical immune checkpoint molecule that inhibits T-cell activation, and its downregulation could potentially enhance antitumor immune responses. In contrast, VISTA mRNA expression remained unchanged following 13F3 treatment. This differential regulation highlights the specificity of 13F3 in targeting certain immune checkpoint pathways while sparing others.

### Antimouse VISTA neutralizing antibody (13F3) mitigates histopathological hallmarks of iARDS-induced diffuse alveolar damage

**Figure 8:**
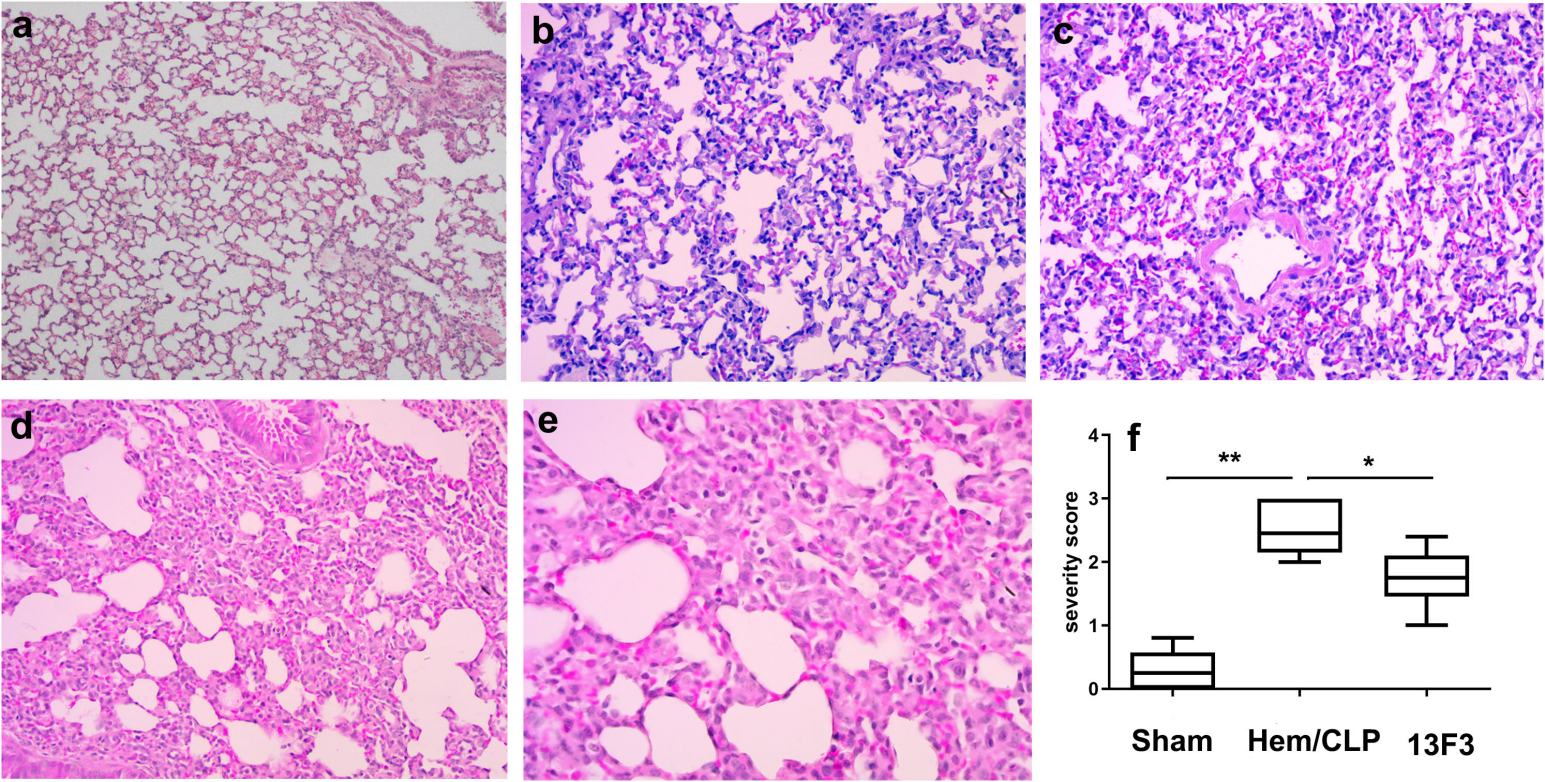
13F3 mitigates histopathological hallmarks of iARDS-induced diffuse alveolar damage. Representative H&E-stained lung sections from iARDS mice demonstrate severe injury characterized by alveolar wall thickening, edema, hyaline membrane formation, macrophage infiltration, alveolar collapse, hemorrhage, and epithelial disruption. 13F3 treatment markedly attenuated these features, reducing edema and inflammatory cell influx while preserving alveolar architecture. Quantification of lung injury severity scores (*inset*) confirmed significant improvement in 13F3-treated groups, highlighting its therapeutic efficacy in countering ARDS-driven tissue damage. **(a)** Sham (WT, × 200 magnification), **(b)** Hem/CLP (WT, × 200 magnification), **(c)** Hem/CLP (WT, × 400 magnification), **(d)** Hem/CLP (13F3 treated, × 200 magnification), **(e)** Hem/CLP (13F3 treated, × 400 magnification), **(f)** severity scores in different groups. The data in **(f)** are presented as the mean ± SEM. *p*-values were determined by the Kruskal–Wallis test. ^*^ *p* < 0.05; ^**^ *p* < 0.01.

Under an optical microscope, iARDS lung injury was characterized by alveolar wall thickening, alveolar edema, hyaline membrane formation, macrophage cell infiltration, alveolar collapse, hemorrhage, and epithelial disruption. These features were collectively demonstrated in the diffuse alveolar damage, which was attenuated by 13F3 (*p* < 0.05, **Figure 8**).

## Discussion

The present study provides novel insights into the role of VISTA in modulating the pathophysiology of iARDS. Our findings demonstrate that VISTA deficiency exacerbates lung injury and mortality in iARDS, while antibody-mediated targeting of VISTA, with 13F3, alters inflammatory mediator profiles across systemic and local compartments. Collectively, these results suggest that VISTA serves as a critical regulator of inflammatory responses in iARDS, potentially offering a new therapeutic avenue for this life-threatening condition.

We found the “double-hit” mouse model could induce iARDS completely in the previous study (20) which simulated shock patients followed by sepsis clinically. The CTLA-4/B7 and PD-1/PD-L1 are two of the most representative immune checkpoint mechanisms that negatively regulate T-cell function during different phases of T-cell activation (24, 25). However, current literature reports that only a small percentage of patients receiving anti-CTLA-4/B7 or anti-PD1/PD-L1 therapies experience prolonged survival (26). As a member of the B7 family, VISTA expression in the hematopoietic compartment varies depending on cell type, maturation stage, tissue location, and species (27).

As we previously reported, frequent expression of VISTA and PD-(L)1 in iARDS (20) suggests that VISTA could be a novel therapeutic target in iARDS, similar to anti-PD-(L)1 immunotherapy (28). While 13F3 did not suppress VISTA mRNA, it did reduce PD-L1 expression in the lung. Gao et al. demonstrated that nuclear PD-L1 can stimulate NF-κB pathway activity and regulate VISTA expression (29). Furthermore, combined blockade of VISTA and PD-1 has been shown to significantly reduce tumor growth (30–32). Unlike VISTA, the immune checkpoint proteins CTLA-4 and PD-1 do not directly regulate myeloid cell function (33). We hypothesize that the function/expression of VISTA and PD-L1 may partially overlap. Similar to other B7 family members, VISTA acts as a negative immune checkpoint regulator, suppressing T-cell immunity. In VISTA-deficient mice, chemokines such as MCP-1 and TNF-α were elevated, while the anti-inflammatory cytokine IL-10 was systematically suppressed. Although cytokine and chemokine levels remained unchanged in VISTA-deficient mice compared to wild-type mice in iARDS, it can be inferred that the local inflammatory response was in dynamic equilibrium, ultimately leading to release into the systemic circulation.

The severe lung injury and reduced survival observed in VISTA^−/−^ mice during iARDS highlight the protective role of VISTA in this model. This finding aligns with growing evidence that immune checkpoint molecules, including VISTA, help regulate hyperinflammatory responses in acute lung injury (ALI) and sepsis (15, 34). The elevated plasma levels of IL-6, IL-10, MIP-2, and KC in VISTA^−/−^ mice suggest a dysregulated cytokine/chemokine response in the absence of VISTA. While IL-6 and KC are classically associated with neutrophil recruitment and proinflammatory cascades (1), the concurrent rise in anti-inflammatory IL-10 highlights a compensatory mechanism aimed at counterbalancing systemic inflammation. The compartment-specific differences in mediator levels (e.g., stable lung tissue vs. altered peritoneal fluid) further underscore the organ-specific roles of VISTA in regulating inflammation, possibly through differential engagement with myeloid and stromal cells.

The 13F3 anti-VISTA monoclonal antibody treatment significantly reduced VISTA expression on monocytes, neutrophils, macrophages, and structural cells (epithelial/endothelial), consistent with findings in cancer models where VISTA blockade enhances immune activation (27). However, in our iARDS model, this intervention only partially modulated cytokine/chemokine profiles. Specifically, systemic (plasma) and peritoneal fluid mediators exhibited dynamic changes, whereas lung tissue mediator levels remained relatively stable. This divergence may reflect distinct roles for VISTA in regulating local vs. systemic immune responses, with lung-resident cells (e.g., alveolar macrophages) potentially relying on alternative checkpoint pathways to maintain tissue homeostasis. The partial efficacy of the 13F3 anti-VISTA antibody—evidenced by reduced VISTA expression but only modest changes in mediator profiles—suggests that VISTA’s protective effects may involve noncytokine mechanisms, such as cellular adhesion, apoptosis, or metabolic reprogramming. This duality underscores the complexity of targeting immune checkpoints in acute inflammation, where their roles differ fundamentally from those in chronic conditions such as cancer (35, 36). Notably, epithelial cells also play an essential role beyond serving as a physical barrier. They can produce antimicrobial peptides and secrete various mediators that modulate immune responses and immune functions (37).

The pathogenesis of iARDS is characterized by dysregulated systemic inflammation, often triggered by extrapulmonary insults such as sepsis, trauma, or pancreatitis (1, 38). In this study, we demonstrate for the first time that VISTA, an immune checkpoint molecule, plays a critical protective role in mitigating lung injury and mortality in iARDS. Our findings show that genetic deletion of VISTA exacerbates disease severity, whereas therapeutic targeting of VISTA modulates inflammatory mediator profiles, underscoring its potential as a novel immunomodulatory target in this devastating syndrome. The exacerbated lung injury and reduced survival observed in VISTA^−/−^ mice align with emerging evidence that immune checkpoints are essential for maintaining immune homeostasis during acute inflammatory syndromes. Unlike its role in cancer, where VISTA promotes tumor immune evasion by suppressing antitumor T cells (39), our data suggest that, in iARDS, VISTA functions as a brake on excessive inflammation. This protective role is supported by the elevated systemic levels of proinflammatory mediators (IL-6, MIP-2, KC) and the compensatory anti-inflammatory IL–10 observed in VISTA^−/−^ mice. The surge in IL-6 and neutrophil-recruiting chemokines (MIP-2, KC) likely contributes to neutrophil infiltration and tissue damage in the lungs, whereas the concurrent rise in IL-10 may reflect a failed compensatory mechanism to counterbalance uncontrolled inflammation in the absence of VISTA. These findings are consistent with studies in sepsis, where immune checkpoints such as PD-1 and T cell immunoglobulin and mucin domain-containing molecule 3 modulate cytokine storms to prevent organ damage (40, 41). A striking observation in our study is the compartmentalized effects of VISTA: while plasma and peritoneal fluid showed dynamic changes in cytokine/chemokine levels, mediator levels in lung tissue remained relatively stable despite the presence of severe injury. These findings suggest that VISTA’s protective role in iARDS may operate primarily at the systemic level, potentially through the regulation of myeloid cell activation (e.g., monocytes, neutrophils) prior to their migration into the lungs. The peritoneal cavity—a key site of extrapulmonary injury in iARDS models—exhibited altered mediator profiles in VISTA^−/−^ mice, indicating that VISTA may modulate early inflammatory cascades at the site of initial insult. This compartment-specific regulation mirrors observations in acute lung injury models, where immune checkpoints are known to differentially modulate local vs. systemic immune responses. Our data support a model in which VISTA restrains excessive inflammation during iARDS, potentially by suppressing myeloid cell hyperactivation or limiting T-cell-driven immunopathology. This role contrasts with VISTA’s established role in cancer, where it promotes immune evasion (42), but aligns with studies demonstrating that immune checkpoints such as PD-1/PD-L1 can mitigate sepsis-induced organ damage (43, 44). The elevation of IL-10 in VISTA^−/−^ mice, a cytokine known to suppress macrophage activation and cytokine production in innate immune cell types (45), may represent a failed compensatory attempt to control inflammation in the absence of VISTA’s regulatory function. Notably, IL-10 plays a key role in regulating the duration of inflammation (46). The observed discordance between plasma and tissue mediator levels further highlights the importance of understanding compartment-specific immune regulation in ARDS pathogenesis.

The protective phenotype of VISTA in iARDS positions it as a promising therapeutic target. However, the partial efficacy observed with 13F3 treatment suggests that factors such as timing, dosage, or combination therapies may be crucial for successful clinical translation. For example, early VISTA agonism (to suppress hyperinflammation) followed by later blockade (to resolve immunosuppression) could parallel effective strategies employed in sepsis (15). It is worth noting that VISTA exerts dynamic effects at various levels of immune cell regulation and disease contexts which may also explain partial protective activity seen with 13F3 treatment. For example, in autoimmune uveitis, VISTA’s constitutive expression in retinal outer segments decreases during disease peaks, suggesting a protective role in patients with this condition (47). In cancer, MEK inhibition combined with PD-L1 blockade reduces CD8^+^VISTA^+^ T cells, correlating with poorer survival in biliary tract cancer patients (48), underscoring context-dependent effects. Furthermore, recent subcellular studies reveal that VISTA is vesicularly stored in macrophages and T cells, allowing for rapid mobilization to the cell surface upon immunogenic stimulation (49). Intracellularly, VISTA interacts with galectin-9 to facilitate TAK1 binding, thereby safeguarding lysosomal integrity (50). Additionally, its NPGF motif recruits NUMB and Rab11 to constrain growth receptor signaling and epithelial proliferation (51), effectively suppressing cell proliferation. VISTA expression is also modulated by tumor microenvironment factors like TGF-β and hypoxia (50), highlighting its role in integrating extracellular signals with cell-intrinsic regulatory functions.

Limitations of this study include the lack of mechanistic insight into how VISTA regulates specific cell populations (e.g., neutrophils vs. Tregs) and its interaction with other checkpoints (e.g., PD-1, TIM-3). Additionally, the use of a murine model necessitates validation in human ARDS cohorts, where VISTA expression and function may differ. Future studies are warranted to investigate the cell-specific contributions of VISTA (e.g., conditional knockout models in myeloid vs. T cells), which may help elucidate its underlying mechanisms in iARDS. Secondly, the ligands and downstream signaling pathways engaged by VISTA in iARDS remain to be fully elucidated, as does the therapeutic potential of VISTA agonists (e.g., recombinant proteins) in early-phase ARDS. While it has been inferred that VISTA may bind to P-selectin glycoprotein ligand 1 (PSGL-1) under acidic conditions, such as those found in the TME, this interaction does not occur at neutral pH. Notably, VISTA is a member of the B7 family but possesses only an immunoglobulin variable domain, lacking an immunoglobulin constant domain. The histidine-rich immunoglobulin variable domain of VISTA contains two additional disulfide bonds that stabilize an unusual structure featuring a 21-amino-acid loop. Based on crystal structures and mutagenesis studies, Johnston et al. proposed a model in which a cluster of five histidines interacts with negatively charged sulfated tyrosine and glutamic acid residues on PSGL-1. This interaction is enhanced under acidic conditions, where the histidines are protonated at acidic pH (13). However, in the iARDS model—unlike the TME of solid tumors—VISTA may not interact with PSGL-1. There have been concerns that the clinical utility of VISTA-targeting therapies may be limited by the lack of a well-characterized receptor–ligand interaction, and the antibody’s affinity under acidic pH remains unknown. Through mutagenesis studies, the binding site of the anti-VISTA antibody used in a terminated clinical trial was mapped to a positively charged surface that includes the extended loop and α-helix—features unique to the VISTA structure. This region also mediates binding to V-set and immunoglobulin domain-containing protein 3 (VSIG3); however, VISTA binds weakly to VSIG3, and the interaction is only moderately pH-dependent (52, 53). In addition to PSGL-1 and VSIG3, VSIG8 (27) has also been reported to bind directly to VISTA, contributing to its immunosuppressive functions. These binding partners of VISTA may serve as potential targets for therapeutic intervention; however, their clinical relevance remains unclear. Moreover, we cannot exclude the possibility that the mechanisms of VISTA’s actions in iARDS mirror those observed in the tumor microenvironment. Another area warranting further investigation is the interplay between VISTA and comorbidities (e.g., ongoing viral infections, obesity, smoking, etc.) that predispose individuals to ARDS. Thirdly, there are concerns regarding the specificity and limitations of the anti-VISTA antibody (13F3), particularly its incomplete suppression of inflammation and pH-dependent activity. The 13F3 monoclonal antibody is a well-characterized blocking antibody that binds VISTA and disrupts its immunosuppressive signaling. However, unlike complete checkpoint inhibitors (e.g., anti-PD-1), 13F3 provides only partial suppression of inflammation. At physiological pH (7.4), VISTA adopts a “closed” conformation that favors immunosuppressive interactions with T cells. In acidic microenvironments (pH ≤ 6.0, e.g., tumors, inflamed tissues), VISTA undergoes a conformational shift to an “open” state, potentially altering its binding affinity for 13F3 and other ligands. If 13F3 preferentially recognizes the closed conformation, its inhibitory effects may be reduced in inflamed or hypoxic tissues where the pH is low. The dynamic pH landscape in conditions such as autoimmunity or cancer may result in heterogeneous 13F3 activity, which could explain its partial suppression or inflammation.

## Conclusion

In summary, our findings establish VISTA as a key modulator of inflammatory responses in iARDS, with genetic deletion exacerbating pathology and antibody-mediated targeting partially altering mediator profiles. These results highlight the dual nature of immune checkpoints in acute inflammatory syndromes, where their roles may diverge from those observed in chronic disease contexts such as cancer. Further investigation into VISTA’s mechanisms and therapeutic potential could pave the way for novel immunomodulatory strategies in ARDS.

## Data Availability

The datasets presented in this study can be found in online repositories. The names of the repository/repositories and accession number(s) can be found in the article/supplementary material.
